# Recommendations for the diagnosis and management of Fabry disease in pediatric patients: a document from the Rare Diseases Committee of the Brazilian Society of Nephrology (Comdora-SBN)

**DOI:** 10.1590/2175-8239-JBN-2021-0216

**Published:** 2022-02-25

**Authors:** Maria Helena Vaisbich, Luís Gustavo Modelli de Andrade, Cassiano Augusto Braga Silva, Fellype de Carvalho Barreto

**Affiliations:** 1 Universidade de São Paulo, São Paulo, SP, Brasil.; 2 Universidade Estadual Paulista, São Paulo, SP, Brasil.; 3 Clínica de Nefrologia Senhor do Bonfim, Feira de Santana, BA, Brasil.; 4 Universidade Federal do Paraná, Curitiba, PR, Brasil.

**Keywords:** Fabry Disease, Consensus, Rare Diseases, Doença de Fabry, Consenso, Doenças Raras

## Abstract

Fabry disease (FD) is a genetic disease, with X-chromosome linked inheritance, due to variants in the GLA gene that encodes the α-galactosidase A (α-GAL) enzyme. The purpose of the present study was to create a consensus aiming to standardize the recommendations regarding the renal involvement of FD with guidelines on the diagnosis, screening, and treatment of pediatric patients. This consensus is an initiative of the Rare Diseases Committee (Comdora) of the Brazilian Society of Nephrology (SBN). Randomized controlled clinical studies and studies with real-life data added to the authors' experience were considered for this review. The result of this consensus was to help manage patient and physician expectations regarding treatment outcomes. Thus, this consensus document recommends the investigation of the pediatric family members of an index case, as well as cases with suggestive clinical signs. From the diagnosis, assess all possible FD impairments and grade through scales. From an extensive review of the literature including pediatric protocols and particularly evaluating pediatric cases from general studies, it can be concluded that the benefits of early treatment are great, especially in terms of neuropathic pain and renal impairment parameters and outweigh the possible adverse effects that were mainly manifested by infusion reactions.

## Resumo

A doença de Fabry (DF) é uma doença genética, com herança ligada ao cromossomo X, devido a variantes no gene GLA que codifica a enzima α-galactosidase A (α-GAL). O propósito do presente estudo foi criar um consenso objetivando padronizar as recomendações em relação ao acometimento renal da DF com orientações sobre o diagnóstico, rastreamento, e tratamento de pacientes pediátricos. Esse consenso é uma iniciativa do Comitê de Doenças Raras (Comdora) da Sociedade Brasileira de Nefrologia (SBN). Foram considerados para esta revisão estudos clínicos controlados randomizados e estudos com dados de vida real somados à experiência dos autores. O resultado desse consenso foi auxiliar no gerenciamento das expectativas de pacientes e médicos quanto aos resultados do tratamento. Assim, este documento de consenso recomenda a investigação dos familiares pediátricos de um caso índice, assim como de casos com clínica sugestiva. A partir do diagnóstico, avaliar todos os possíveis acometimentos da DF e graduar através de escalas. A partir de uma revisão extensa da literatura incluindo protocolos pediátricos e avaliando particularmente os casos pediátricos de estudos gerais, pode-se concluir que os benefícios do tratamento precoce são grandes, principalmente quanto aos parâmetros de dor neuropática e do acometimento renal, e suplantam os possíveis adversos que foram sobretudo manifestados por reações infusionais.

## Introduction

Fabry disease (FD; OMIN#301500) is an X-linked, genetic, lysosomal-storage disease caused by pathogenic variants in the GLA gene, responsible for encoding the alpha-galactosidase A (α-GAL) enzyme, involved in the glycosphingolipid’s metabolism. The deficiency or absence of an α-GAL action determines intralysosomal buildup of globotriaosylceramide (GL3) and its derivatives, such as its deacylated form, globotriaosylsphingosine (lyso-GL3)^1,2,3^.

SCD is multisystemic, but among the most impacted organs, the kidneys, heart, central (CNS) and peripheral nervous systems^1,2,3,4^ stand out. The possibility of a specific treatment with enzyme replacement therapy (ERT) changed the course of this disease^1,2,4^.

In general, the most serious complications were initially reported between the 3^rd^ and 5^th^ decades of life, and that is why FD was considered an adult disease^1^. However, with the new information concerning the FD’s natural history, we know that the pathophysiological process and organ damage start early in childhood and affect both boys and girls^1,2,3^.

Historically, there has been a paradigm shift in relation to FD. The strategy was to detect patients with the clinical impact of the disease, such as by screening patients on dialysis for an unknown cause^4^; however, the objective is being expanded to detect younger patients, oligo or asymptomatic, identified by family screening, in which early diagnosis and treatment can mitigate or avoid the complications of the disease^5^. Among the factors responsible for this change, there is evidence of early childhood involvement, both with general symptoms and signs of FD nephropathy.

The creation of a consensus concerning FD recommendations in Pediatrics is limited by the small number of cases in the studies, including in the registries^2,3^, lack of impactful studies and validated biomarkers that reflect the “pre-symptomatic” progression of the disease. However, considering the new evidence, it is urgent to create a document of recommendations regarding the conduct for FD in the pediatric age group.

Therefore, upon creating this document, Comdora intends to disseminate information about Pediatric FD and suggest some approaches for the diagnosis and management of these cases, with a main focus on FD Nephropathy.

## Objectives of the brazilian pediatric consensus on fabry’s disease (Comdora-SBN)

This consensus is an initiative of the Comdora - SBN, aiming to standardize the recommendations regarding the renal involvement in FD with guidelines on the diagnosis and treatment of pediatric patients.

## Methods used in making these recommendations

We formed a Brazilian expert panel with the aim of developing a diagnostic and therapeutic consensus for FD in pediatrics, based on expert opinion, and a systematic review of the literature. We performed a systematic literature review by electronically searching in Medline, PubMed, and the Cochrane Library using the search terms “Fabry” and “Fabry disease” and “pediatric/children” without language restrictions up to the date of June 2021.

Based on literature recommendations for rare diseases, we considered methodologically less rigorous studies that include real-life data. Therefore, we considered case series, cohort studies and registry studies. In addition, the authors’ experiences were considered, especially in points that are still controversial.

The systematic review of the literature and the meetings of the Brazilian expert panel were carried out by the Comdora group. This paper presents the consensus reached on the therapeutic goals developed by specialized working groups, responsible for developing therapeutic goals, mainly for the kidney, in addition to a consensus on the goals for the treatment of other systemic manifestations of FD with a focus on pediatrics.

Throughout the text, the classes of evidence and recommendations will be used as summarized in Table 1, subdivided into class I (recommended), class II (potentially recommendable) and class III (not recommended)^29^.

**Table 1:** Clinical characteristics of patients with Fabry disease (FD), highlighting the median (or average) age and the lowest age of symptom onset (+ early), according to sex, in addition to the possibilities of differential diagnosis

Signs / symptoms	Frequency % total; ♂%;♀%	Median Onset: years (a); Earliest age reported (+)	Main differential diagnosis
Pain (dysesthesia); burning spells in hands and feet^9^	50-72% ♂59-67%; ♀40-65%	♂ 7-10 y; ♀ 8-15 y + early: 2-4 y	Growing pain Rheumatologic diseases (fibromyalgia and others).^10^
Hypohidrosis or anhidrosis	25-59% ♂ 28-93% ♀17-25%	♂ 8-10 y; ♀ 4 y + early: 2,5 y	Causes of dysautonomia; usually with other manifestations.
Cornea *verticillata*	50-71,5% ♂ 36-73%; ♀65-70%	♂12 y; ♀ 9 y + early: newborn	Use of hydroxychloroquine or amiodarone.^11^
Gastrointestinal symptoms	18-50% ♂ 23-40%; ♀ 11-20%	♂5 y; ♀ 9,5 y + early: 1 y 4 y	Irritable bowel syndrome, food intolerances^12^
Intolerance to exercise/heat/cold	17-39% ♂ 17-39%; ♀ 17-38%	♂5-7y; ♀ 8-16 y + early: 3,5 y	Disorders of muscle channels of Ca^++^ and K^+^.^13^
Angiokeratomas	14-40% ♂ 20-57%; ♀ 8-38%	♂7-9 y; ♀9,5-14 y	Some deposit diseases. In FD, it usually occurs, but not only, in the region of the swimming trunks, posterior face of the buttocks and thighs and, periumbilical.^14^
Hearing loss	19-22% ♂19%; ♀24%	♂2,7 y; ♀ 14,4 y + early: 4 y	Other disorders with sensorineural deafness that have other manifestations.^15^
Kidney changes - Hyperfiltration - Microalbuminuria - Proteinuria	- ??? - 13-16%^4,5^ - 14-20%^4,5^	- ??? -♂ 16 y; ♀ 16 y -♂ 14 y; ♀ 14 y	Causes of proteinuria without SN^16^. HF+ is an impactful data for the diagnosis of FD. Do not hesitate to have a kidney biopsy.
Heart alterations - conduction - Valve disfunction - Arrhythmias - LVH	- 8%; ♂8-10%; ♀4-7%^6^ - 15-18%; ♂6-23%; ♀ 14-24%^6^ - 1-5%; ♂3-7%; ♀ 0-2,5%^6^ - 3/22 (13,6%) children with DF^6^	- ♂ 10 y;♀ 17 y - ♂ 8,6 y;♀ 14 y - ♂ 9,3 y - ???	Other causes of these alterations^16^. Positive family history is an important data for the diagnosis
CNS involvement - Rare in pediatrics; usually in Young adults.^17^	MRI in FD in pediatrics (mean age, 14 years) versus controls:17 Asymptomatic white matter lesion: 16% versus 6.5% 91% of patients already had neuropathic pain, cornea *verticillata* and/or abdominal pain. No cases with CKD, heart disease or high blood pressure. Positive family history is an important data for the diagnosis.		

## FD diagnosis in pediatrics

### Pediatric patient identification by family screening

**Figure 1:**
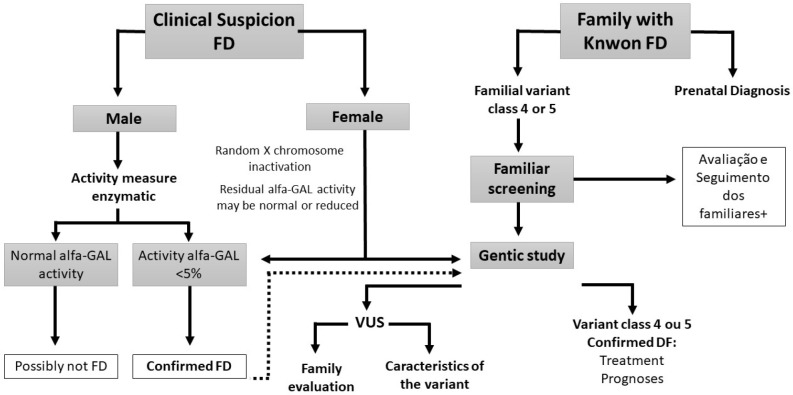
Flowchart for identification and diagnostic investigation of Fabry disease.

We recommend the investigation of all pediatric family members of an index case, after discussion and in agreement with family members. A detailed clinical history, added to a detailed physical examination, can detect pediatric patients with incipient clinical signs. The screening methods used in pediatric patients should be the same used for adults^6^, being essential in men to measurement the α-GAL activity and genetic analysis in women, as shown in Figure 1. Caregivers of asymptomatic children should be instructed concerning the appearance of symptoms and to seek medical attention promptly.

Caption: XCi= degree of x chromosome inactivation between cells.

### Diagnosis of fd in pediatrics from clinical suspension

The diagnosis of FD is infrequent in pediatrics due to the nonspecific nature of the initial symptoms. An interval of 13.7 years between the onset of symptoms and diagnosis was detected in men and of 16.3 years in women^7^, and the frequency of misdiagnosis is approximately 25%^8^.

The suspicion of FD arises from the clinical signs and symptoms, excluding other more common pediatric causes, and from family history. Table 1 shows some of the FD characteristics and the data reported in Pediatrics.

FD nephropathy is insidious and progressive^18^, and more than half of men and about 20% of women develop advanced chronic kidney disease (CKD)^19^. Considering this situation, research seeks to identify the initial lesions and indicate early treatment, aiming to slow down the progression of CKD and prevent cardiovascular and CNS complications. Kidney injuries result from the deposition of GL3 in kidney cells, which triggers a series of events, culminating in cell death and tissue fibrosis^20^. Table 2 shows the main studies that detected renal alterations in FD in Pediatrics reviewed for the creation of these recommendations.

**Table 2:** Studies showing early kidney changes in SCD in Pediatrics

Author	Year	Patients	Findings
Elleder M et al.^21^	1998	fetus	GL3 inclusions, mainly in fetal podocytes.
Tøndel et al.^22^	2008	9 children (7 to 18 years) with normal GFR and minimal or no proteinuria	GL3 inclusions in podocytes and distal tubules accompanied by fusion of the pedicels processes in all cases, arteriolopathy in almost 50% of patients and focal segmental glomerulosclerosis (FSGS) in adolescents.
Ramaswami U et al.^23^	2010	children	Renal histological changes before microalbuminuria/proteinuria.
Najafian et al.^24^	2013	children	An increase in podocyte GL3 correlates with foot processes fusion and albuminuria.
Branton et al.^25^	2002	Not reported	There was a faster decrease in GFR in patients with higher initial proteinuria, corroborating the understanding that proteinuria is a late indicator of FD nephropathy.
Riccio E et al.^26^	2019	children	Initial kidney lesions in younger patients are earlier translated into glomerular hyperfiltration and are often detected before the onset of microalbuminuria, usually associated with mild or no extrarenal symptoms. There was negative correlation between GFR and age, GFR and levels of proteinuria and GFR and cardiovascular manifestations.
Trimarchi H et al.^27^	2015	patients	Podocyturia in FD patients precedes microalbuminuria.
Politei et al.^28^	2018	children (4 to 9 years)	They found increased plasma lyso-GL3 and podocyturia in all of them, but still with normal estimated GFR (eGFR) and only half of them had microalbuminuria. The kidney histology of these patients revealed glomerular, interstitial and vascular changes.

Evaluating these studies, it is possible to conclude that: (I) FD nephropathy begins during intrauterine life and that markers such as microalbuminuria/proteinuria, previously considered early, proved to be late^29^; (II) renal changes sequentially include: GL3 deposits, detachment of podocytes from the glomerular basement membrane (GBM) and podocyturia, denudation of the GBM and effacement (enlargement) of podocyte pedicels, glomerular hyperfiltration, followed by microalbuminuria and proteinuria. Decreased glomerular filtration rate (GFR) occurs in later stages of the disease. Patients can still develop secondary nephrogenic diabetes insipidus, hypokalemia and renal tubular acidosis due to GL3 deposits in collecting tubule cells^30^.

### Diagnostic confirmation

The diagnostic criteria in Pediatrics are similar to those for adults and can be seen in Table 3. Some particularities in Pediatrics include:

**Table 3:** Criteria for the diagnosis of FD

Men		Women	
Having the genetic variant		Having the genetic variant	
+		+	
α-GAL deficiency ≤ 5%		Not necessary to measure α-GAL	
+			
**A or B or C or D #**			
**A (clinical)** Having 1 or more factors: neuropathic pain, cornea verticillata or angiokeratoma	**B (biochemical)** Elevated plasma or urinary GL3 or lyso-GL3 (> 1.8ng/ml)	**C (familiar)** Family member with a definitive diagnosis of FD carrying the same variant	**D (histological)** Histological changes suggestive of lysosomal deposits in target organs (kidneys, skin, heart)

Legend: FD (Fabry disease), α-GAL (α-galactosidase A), GL3 (globotriaosylceramide), lyso-GL3 (globotriaosylsphingosine).

# Exception: men with pathogenic mutation (class I) and α-GAL activity ≤ 5%, but without other criteria (A/B/C/D).

- **Genetic testing in children born to parents with FD:** recommended by the European Society of Human Genetics, it offers the possibility of early diagnosis and treatment, if necessary^14^.

- **Prenatal diagnosis**: in some countries, legislation allows pre-implantation diagnosis and embryo selection in cases of assisted reproduction. It may be indicated in families with known FD^8^.

- **Renal biopsy**: it is not considered essential for the diagnosis, and its indication in FD must be judicious. Despite being safe, when performed under appropriate conditions, it is not routinely indicated in our environment; however, in some clinics in North America and Norway, renal histological analysis is part of the investigative routine of children with FD^22^. It is an essential tool in inconclusive cases with high clinical suspicion, such as in cases of variants of uncertain significance (VUS), in which there are no family members available for investigation, a situation in which the identification of tissue deposit of GL3 is of paramount importance for diagnosis confirmation. Renal biopsy may also be indicated in cases with a pathogenic variant, family history of severe FD nephropathy, progressive increase in plasma lyso-GL3 and absence of symptoms, and to indicate initiation of RRT. In these cases, the presence of GL3 inclusions in the renal cells can be observed and associated with histological alterations, such as the effacement (enlargement) of the podocyte pedicels, and other lesions that demonstrate the histopathological evolution of the disease, up to the presence of segmental and focal glomerulosclerosis. (FSGS) in more advanced cases.

- **Skin biopsy**: in practice, confirmation of FD can be done through skin biopsy; it is a simple, well-tolerated procedure that provides unequivocal evidence of loss of fine fibers, being useful for the differential diagnosis, especially in patients with no family history.

## Management of pediatric patients with FD

Pediatric management of FD is different from the procedure in adults. The search is for incipient markers of disease onset, preferably its progression. They are “pre-symptomatic”, which can provide criteria for the initiation of specific treatment^2,3^.

In this age group, the use of validated scales for monitoring is essential, both in the pre-symptomatic and symptomatic phases, as well as for detecting changes with treatment and comparing different cohorts. Specific scales and general scales adapted for FD can be used; for example, for the evaluation of neuropathic pain, the Brief Pain Inventory (BPI) has been used with good results^32^.

The standardized evaluation script must include a thorough investigation of the symptoms and signs of involvement of the different organs and systems involved^8^.

In the literature, there are some recommendations for pediatric FD in asymptomatic patients; for example, that a complete evaluation (clinical and laboratory) should be performed at the time of diagnosis, and at least annually in boys and every 2 years in girls^8,33^. Our recommendation is that the proposed interval be individualized, regardless of gender, according to the severity of the evolution of the affected family members.

### Assessment of FD signs and symptoms - focus on pediatrics

Table 4 shows the general recommendations for monitoring pediatric patients diagnosed with FD. Renal involvement will be detailed here because it is early in childhood and has a great impact on the patient.

**Table 4:** Monitoring of asymptomatic and symptomatic cases and patients undergoing treatment

EVALUATION INTERVAL	
Baseline (upon diagnosis/ERT onset)	All the cases
sequential OBS.: In symptomatic cases and under ERT, the intervals should be reduced according to the needs.	Boys, at least annually (asymptomatic). Girls, at least every 2 years (asymptomatic).
OVERAL CLINICAL ASSESSMENT OF THE PATIENT	
General physical examination data, examples.	Adequate assessment of anthropometric data. Adequate measurement of blood pressure in the office or ABPM in selected cases.
GENERAL CLINICAL EVALUATION OF FD - PREFERENTIALLY USE SCALES	
General scales, examples	Pain Scales: Brief Pain Inventory (BPI) Scale for GI Symptoms: Gastrointestinal Symptom Rating Scaled
Specific scales for Fabry disease, examples	MSSI, DS3, Fabry Stabilization index - FASTEx
GENERAL LABORATORIAL ASESSMENT	
FD-related laboratory tests	At least annual serum Lyso-GL3 (DBS)
Monitoring the general situation of the patient according to needs, examples.	Lipid profile, uric acid and blood glucose assessment.
SPECIFIC LABORATORIAL ASESSMENT	
Kidney function assessment	Albuminuria, tubular dysfunction, eGFR (Schwartz, Modified Schwartz, CKD-EPI), measured GFR (24-hour urine creatinine clearance, radioisotopic methods in selected cases)
Heart assessment	EKG, Doppler echocardiogram (preferably with strain), cardiac MRI - not routine in Pediatrics; perform exceptionally in selected cases.

- **Initial assessment and monitoring of renal involvement:** The kidney is among the earliest and most severely affected organs. Thus, attention should be paid to minor functional changes and treatment should be initiated, avoiding or mitigating the serious outcome of FD nephropathy.

- **Assessment of glomerular renal function:** in practice, measurement of GFR calculated from 24-hour urine is recommended at diagnosis and treatment initiation. If it is not possible to collect timed urine, it is recommended to use pediatric equations to estimate renal function, which are more accurate than isolated serum creatinine^34^. The important thing is to use the correct formulas, according to sex and age, and with the proper interpretation^34-39^. The combined equation for calculating GFR based on serum cystatin C and creatinine is currently considered to be the most accurate for estimating GFR in children34. However, cystatin C is not routinely available in many facilities.

In the follow-up, the formulas can be used and, exceptionally, the 24-hour urine creatinine clearance can be calculated. Radioisotopic methods, such as Cr51 EDTA clearance^34,40^, are indicated in selected cases, for example, when there is doubt about the presence of glomerular hyperfiltration, which may be an indication criterion for initiation of RRT.

**- Assessment of tubular functions:** the presence of tubular dysfunction should be assessed by FD, which can be performed by analyzing venous blood gas analysis, serum and urinary electrolytes, urinary density and osmolality.

**- Examination of urine and urinary sediment:** to check for microalbuminuria/proteinuria and changes in urine concentration, isosthenuria or hyposthenuria^30^. Hematuria is rarely observed in these patients and its presence should alert for the presence of other diseases.

**- Proteinuria, microalbuminuria and creatinine in 24-hour urine:** indicated at diagnosis and annually at follow-up. The measurement of urinary creatinine serves to calculate the GFR and ensure that the 24-hour collection is adequate.

**- Proteinuria, microalbuminuria and creatinine in an isolated urine sample:** indicated in cases where timed urine collection is difficult and in the follow-up after treatment onset.

**- Low molecular weight proteinuria in an isolated urine sample:** when available, it can help in the assessment of tubulointerstitial involvement; examples: ß2 microglobulin or retinol-binding protein (RBP)^41,42^.

**- Kidney and urinary tract ultrasound:** indicated at diagnosis and annually during follow-up.

**- Analysis of renal histology (renal biopsy):** indicated when there is diagnostic doubt, in the staging of the initial kidney injury (in some protocols) and to support the indication of treatment. It is essential to inform the pathologist of the clinical suspicion. We recommend performing a renal biopsy: (a) in the presence of glomerular hyperfiltration, albuminuria/proteinuria, or tubular involvement; (b) to ward off other diseases; (c) in cases without clinical manifestation, but with pathogenic variants, family history of early nephropathy and progressive increase in lyso-GL3. In these cases, waiting for the appearance of proteinuria/microalbuminuria may delay the initiation of treatment.

Among the relevant findings are the signs of arteriolopathy and the detection of GL3 deposits in renal cells, especially in podocytes, and their histological repercussions^22^.

Although the reduction of deposits and histological improvement have been demonstrated in a study of sequential biopsies in children^43^, we do not recommend that this be a routine parameter. Re-biopsy in FD is recommended in cases of sudden worsening of renal function, inadequate response to treatment and progression of FD nephropathy, for differential diagnosis with other diseases or to evaluate the change of specific therapy.

Currently, the earliest available method for detecting renal involvement is histology^22,43^.

**- Sequential markers of renal involvement by FD:** in Pediatrics, the use of non-invasive indicators is recommended to identify renal disease and its progression, such as GFR, urinary concentration, proteinuria/microalbuminuria, venous gases and electrolytes. In the future, an earlier and non-invasive marker is expected, such as podocyturia.

**- Assessment of other factors:** it is recommended to monitor other risk factors involved in the progression of CKD, such as systemic arterial hypertension (SAH) and dyslipidemia^44,45^. Appropriate blood pressure measurement is indicated at all visits and should be kept below the 90th percentile for the patient’s age, sex, and height. We recommend performing outpatient monitoring of blood pressure (ABPM) in patients over 5 years of age with suspected white coat or masked hypertension^46^.

### Metabolic monitoring - biomarkers

Plasma lyso-GL3 is considered a biomarker of disease activity and it is recommended for diagnosis and follow-up^47^.

Monitoring in children diagnosed with pathogenic gla variant for non-classic phenotype

Monitoring should focus on the affected organ (heart or kidney) and its frequency should be determined on a case-by-case basis^48^.

## FD treatment in pediatrics

### Recommendations for starting specific treatment

In Pediatrics, the greatest peculiarity is the moment when specific treatment is indicated. The goal of specific treatment is to be early enough to limit or prevent irreversible tissue damage and minimize disease symptoms, balancing the risk of side effects and discomfort from medicalization.

Specific treatments in Pediatrics include the two enzymes indicated for RRT and approved by ANVISA, agalsidase alfa (ALFA) and agalsidase beta (BETA), approved from 7 and 8 years of age, respectively. The oral chaperone, migalastat, is also available, approved from the age of 16, but its use is restricted to patients with missense mutations susceptible (amenable) to this drug^49^.

The largest body of evidence in Pediatrics is based on RRT, which will be referred to in these recommendations. Supplementary Table 1 shows a review of studies with RRT that included pediatric cases of FD, with 19 publications using ALFA, 15 using BETA, 6 jointly evaluating ALFA and BETA, and 5 studies using unspecified RRT.

However, there are 18 case report studies, considering a case report study with the description of up to 3 patients, and only 21 studies were designed specifically for the pediatric population, including cohorts and registry studies. As a result of these studies, it can be said that RRT in children is safe and well tolerated. However, when indicating treatment, benefits and unwanted effects must be balanced. Among the benefits found in the studies (Supplementary Table 1) the clinical improvement of neuropathic pain, gastrointestinal symptoms and heat intolerance stand out. Considering the pediatric studies, the potential benefit of mitigating or preventing serious renal outcomes observed the earlier the RRT was instituted is highlighted, including normalization of GFR in patients who previously had hyperfiltration, GFR stability and reversal of microalbuminuria/proteinuria. Regarding cardiac involvement, the few studies report that the ideal is to start RRT before this involvement, as the response may not be as satisfactory as that observed in relation to renal prognosis.

The only study that evaluated the very early initiation of RRT, that is, at the stage in which patients were practically asymptomatic, did not detect significant changes at follow-up, but the patients were almost asymptomatic at baseline^80^. In this case, the fact that there were no changes would be a positive point in the early initiation of RRT, that is, in the pre-symptomatic phase.

Another interesting finding in these studies was the small interference of IgG antibodies on disease outcomes.

There were infusion reactions with the use of BETA, but some patients using ALFA had occasional reactions, especially at the beginning of its use.

We did not find studies that evaluated outcomes associated with the emotional and social impact of medicalization and the need for repeated venous punctures or long-term catheters.

There is no universal consensus concerning the indication of RRT onset in children. Based on published recommendations^8,33^ and on the results of pediatric studies in this review, we present some recommendations on the initiation of RRT in Pediatrics.

**- Recommendations for symptomatic pediatric patients**

They should initiate RRT regardless of sex and even in the presence of mild symptoms^93^.

Regarding the indication for renal involvement, in Pediatrics these indications differ from those of adults. Studies indicate that treatment is recommended in the presence of podocyte injury before the critical loss of these cells^94^. Therefore, renal biopsy may help to decide whether to initiate treatment in pediatric patients - which may be indicated in the presence of GL3 deposits in renal tissue.

**- Recommendations for asymptomatic patients**

In patients with a confirmed diagnosis of FD, but asymptomatic, the indication of initiation of RRT has been the subject of discussion.

In boys with classic mutation, RRT is indicated from 7 or 8 years of age^5^. This indication is based on renal biopsy and RRT response studies^10,94,95^. This consensus agrees with that recommendation. However, some authors still recommend starting RRT above 16 years of age^9^, while others consider starting RRT in asymptomatic boys with a pathogenic GLA variant, family history of severe disease in men, undetectable α-GAL activity and plasma lyso-GL3 > 20 nmol/L^8^.

The initiation of treatment in boys under 7 years of age is still a matter of great debate. Possibly, a subgroup of these patients who present the coexistence of potential risk factors for developing more severe forms of the disease, such as the presence of a classic variant, very reduced or absent enzyme activity, plasma levels of very high lyso-GL3 levels and a family history of severe FD, may benefit from starting RRT at an earlier stage. Such patients could be included in the indications for histological renal evaluation to support the therapeutic discussion. However, as there are no studies with patients in this age group, it is currently not possible to make any recommendation regarding the initiation of RRT in these cases.

In asymptomatic girls, there are no data to support initiation of RRT; however, depending on the severity of the mutation in the family, a progressive increase in plasma lyso-GL3 or if there is a shift in favor of the expression of the mutant GLA allele in the X-chromosome inactivation test (a technique not available in our country), one could consider the beginning of RRT. Knowing that renal tissue involvement may be prior to the increase in plasma lyso-GL3, renal histology could be an even earlier criterion to assist in the decision to start treatment in the case of women, in whom the degree of mosaicism of the accumulation of GL3 is related to podocyte injury, assessed by the enlargement of podocyte processes^11^.

Based on new knowledge about the pathophysiology of FD, it is concluded that the possibility of initiating RRT in children affected by FD nephropathy even before the presence of microalbuminuria would be ideal to prevent more prominent future renal impairment^8,10,94,95^. For this, there is a need for a definitive diagnosis of the disease and the presence of factors, such as increased plasma lyso-GL3, glomerular hyperfiltration, GL3 deposits in renal tissue or indicators of early renal impairment, as it is expected to be podocyturia in the future.

**- Recommendations for patients with non-classical variants**

Patients with non-classical variants (attenuated or late onset) detected from neonatal screening or family screening should be followed up and treatment initiated in the presence of any manifestation, however subtle, of the disease^94^. In these cases, the family must be informed about the expected evolution, and unnecessary procedures must be avoided.

**- Recommendations for patients with VUS**

In the case of patients with VUS detected by neonatal or family screening, the characteristics of the variant and the investigation of family members can help predict the pathogenicity of the variant and contribute to the indication of a more specific test and the beginning of treatment^8^. In cases without the possibility of investigating family members, the ideal would be to carry out a functional study of the variant, but it can be inferred by “*in silico*” prediction instruments and correlate the phenotype with the genotype.

### Which specific treatment for fd to use in pediatrics

Supplementary Table 1 shows an extensive review of studies that included pediatric cases with both commercially available enzymes. In general, the results are positive in both improving general symptoms and preserving vital organ functions. Due to the small number of cases and the inconsistency of the results, there is no possibility of discussing which enzyme is better in this age group. However, for the choice, the possibility of home infusion should be initially considered, which can reduce the problems of medicalization and frequent visits to hospitals or infusion centers. In case of an inadequate response, RRT can be changed and see if the results also improve.

### Supporting treatment

It is recommended to use the therapeutic weapon regularly used to treat CKD, using inhibitors of the renin-angiotensin-aldosterone system for its antiproteinuric, hypotensive and stabilizing effect on glomerular hemodynamics, maintaining control of renal function, serum potassium level and preventing hypotension. Dietary adjustments are indicated to promote adequate weight gain and growth, control the lipid profile and adequate protein-calorie intake. In this document we do not introduce the therapeutic options for the involvement of other organs and systems, but we recommend reading more specific documents.

Monitoring response to treatment

The treatment response should be evaluated in the same way proposed for adults, emphasizing that, as the clinic is more incipient, the earliest parameters of involvement, mainly renal, must be thoroughly investigated^8,33^. The recommendation in most studies is to assess the estimated GFR at least every 6 months during treatment and, in cases of doubt, measure the GFR. Some authors suggest renal biopsy at the beginning of treatment and protocol sequential biopsies to evaluate morphological biomarkers^43^. However, this is not what we recommend in this consensus. In our opinion, re-biopsy should be reserved for cases of sudden worsening of renal function, inadequate response to treatment, progression of Fabry nephropathy, for differential diagnosis with other pathologies or indication of specific therapy change.

For general monitoring, we suggest the use of questionnaires applied to FD, such as the “Main Severity Score Index”, which has also been validated for the pediatric age group^12,13^ or the Fabry-specific Pediatric Health and Pain Questionnaire (FPHPQ)^14^ and the Fabry disease severity scoring system, DS315.

## Conclusions

With more detailed knowledge concerning the natural history of FD, it is known that the mechanisms that determine damage to the affected organs have an early onset, already in Pediatrics. Thus, this consensus document recommends the investigation of pediatric family members of an index case, as well as cases with suggestive clinical signs. From the diagnosis, assess all possible FD impairments and grade through scales. From an extensive review of the literature including pediatric protocols and particularly evaluating pediatric cases from general studies, it can be concluded that the benefits of early treatment are great, especially regarding the parameters of neuropathic pain and renal involvement and outweigh the possible adverse effects that were mainly manifested by infusion reactions. A reliable preclinical biomarker is expected to further support the initiation of treatment, especially in asymptomatic cases.

## Limitations of the study

Most of the studies related to Fabry disease in Pediatrics were performed in cohorts and case series, there is a lack of data from large, controlled studies and, therefore, most of the recommendations of this pediatric consensus are between grades IIA and IIB.

## Supplementary Material

The following online material is available for this article:

Table 1 - Published studies using enzyme replacement therapy for the treatment of Fabry Disease in Pediatric Patients were taken from PubMed. Not only studies designed for the pediatric age group were included, but also pediatric cases selected from studies including patients of various ages. ***Oxford Center for EBM**
