# Supplementary material for: Recommendations for the diagnosis and management of Fabry disease in pediatric patients: a document from the Rare Diseases Committee of the Brazilian Society of Nephrology (Comdora-SBN)
Source: J Bras Nefrol. 2022 Feb 25;44(2):268–80. doi: 10.1590/2175-8239-JBN-2021-0216 (PMC9269176; doi:10.1590/2175-8239-JBN-2021-0216)
Supplement: Supplementary file 1 [file 2175-8239-jbn-2021-0216-suppl1.pdf]

Braz. J. Nephrol. (J. Bras. Nefrol.)
https://doi.org/10.1590/2175-8239-JBN-2021-0216

Supplementary Material for "Recommendations for the diagnosis and management of pediatric patients with Fabry disease - document from the Rare Diseases Committee of the Brazilian Society of Nephrology (Comdora-SBN)."

Table 1 - Published studies employing enzyme replacement therapy for the treatment of Fabry disease in pediatric patients retrieved from PubMed. Not only studies designed for the pediatric age group were included, but also pediatric cases selected from studies including patients of various ages. *Oxford Centre for EBM.

Year	Author (s)	Title	Study type	* Level of evidence	ERT	Patients	Methods	Conclusions
**2003**	Illsinger S et al.[50]	Enzyme replacement therapy in an adolescent with Fabry disease.	Case report	5	ALPHA	Boy 15 years. 20 months of ALPHA ERT.	After 6 months with ALPHA, assessed autonomic symptoms, neuropathic pain (BPI).	↓ pain and autonomic symptoms. 1 case of reaction at the 5th dose (transient pulmonary obstruction and rash).
**2004**	Tümer L et al.[51]	The co-existence of Fabry and celiac diseases: a case report.	Case report	5	BETA	11-year-old girl with FD and celiac disease.	5-year follow-up: proteinuria without ↓ with ACEI. She underwent a renal biopsy and started BETA.	4 months of BETA: ↓ significant proteinuria.
**2004**	Mills K et al.[52]	Monitoring the clinical and biochemical response to enzyme replacement therapy in three children with Fabry disease.	Case series	4	BETA	3 boys (11 to 15 years old) with neuropathic pain.	Assessment: BPI, plasma and urinary GL3. 2-year follow-up.	↓ pain, but GL3 ↑ and 2 off-label dose cases. Significant infusion reactions and ↓ IgG anti BETA titers.
**2006**	Ries M et al.[53]	Enzyme replacement therapy with agalsidase alfa in children with Fabry disease.	Multicentric Open	2	ALPHA	19 boys and 5 girls; median age = 11 years (6.5 to 18).	6 months of ALFA: clinical observation and plasma GL3.	↓ pain, ↓ plasma GL3 and normalized HR. Mild-moderate infusion reactions in 7 cases and transient IgG Ab in 1.
**2007**	Ramaswami U et al.[54]	Enzyme replacement therapy with agalsidase	Cohort	3	ALPHA	9 boys and 4 girls, median age = 11 years (3.5 to 18 years).	23 weeks of alpha. Assessed neuropathic pain (BPI), plasma and urinary GL3.	↓ plasma and urinary GL3 and ↓ neuropathic pain. Adverse events: 11 cases (22% probably related to

Year	Author (s)	Title	Study type	* Level of evidence	ERT	Patients	Methods	Conclusions
		alfa in children with Fabry disease.						ALFA), 1 with ↓ of pain and Ab IgG +.
**2007**	Ries M et al.[55]	Enzyme replacement in Fabry disease: pharmacokinetics and pharmacodynamics of agalsidase alfa in children and adolescents.	Multicentric prospective	1c	ALPHA	19 boys (11.5± 3.8 years) and 5 girls (13.7 ± 4.1 years).	Assessed PK and PD. Comparison with adults.	PK in children similar to that of adults, despite having faster plasma clearance.
**2007**	Hoffmann B et al.[56]	Fabry Outcome Survey European Investigators. Gastrointestinal symptoms in 342 patients with Fabry disease: prevalence and response to enzyme replacement therapy.	Registry data	1c	ALPHA	71 FOS Children	Assessment: GI and QoL symptoms at baseline and after 12 and 24 months of ALPHA.	ALPHA significantly ↓ GI symptoms (abdominal pain and diarrhea), particularly in boys and prevented other symptoms. Time dependent improvement. No improvement in QoL.
**2007**	Gelderman MP et al.[57]	Elevated endothelial microparticles in Fabry children decreased after enzyme replacement therapy.	Letter (Cohort)	1c	ALPHA	10 pediatric patients, 9 boys and 1 girl.	Assessment: the presence of endothelial cell membrane microparticles (EMPs) in the blood before and after 6 and 12 months of ALPHA.	EMPs ↑compared to the control group and significant ↓ with ALPHA. ERT may influence the circulating EMP count in pediatric patients.
**2008**	Wraith JE et al.[58]	Safety and efficacy of enzyme replacement therapy with agalsidase beta: an international, open-label study in pediatric patients with Fabry disease.	Multicentric open	1c	BETA	14 boys (8.5 to 16 years old) and 2 girls (11 years old). GI+ symptoms in 69% of cases, hypohidrosis in 100%, FD pain in 88% and angiokeratoma in 69%.	Evaluation after 12 infusions: GL3 (skin, plasma and urine), renal and cardiac functions, pain, sweat test and QoL.	BETA is safe and ↓ dermal deposits of GL3, ↓ plasma GL3, improves GI symptoms, normalizes GFR in hyperfiltrators and ↓ proteinuria.
**2008**	Tøndel C et al.[22]	Renal biopsy findings in children and adolescents with Fabry disease and minimal albuminuria.	Case series	4	ALPHA	9 cases mean age = 13.5 years (7 to 18) underwent kidney biopsy. 2 patients (16 and 17 years old) had had it for 2 years.	Assessment: GFR (iohexol, Schwartz formula and serum cystatin C); Doppler echocardiogram, EKG and Holter monitoring.	1st systematic renal biopsy study in children. They conclude: Proteinuria was a late marker. They suggest that kidney biopsy should be performed in children before starting ERT. Cardiac changes were rare.
**2008**	Choi JH et al.[59]	Short-term efficacy of enzyme replacement therapy in Korean	Report of 2 cases included in a prospective cohort	4	BETA	2 cases (familial): acroparesthesias, anhidrosis, LVH, cornea verticilatta, GI	BETA from 4 to 27 months, evaluating short-term efficacy.	↓ Plasma, urinary GL3 and GL3 in the glomerular endothelium. Small response about cardiac alterations

Year	Author (s)	Title	Study type	* Level of evidence	ERT	Patients	Methods	Conclusions
		patients with Fabry disease.				symptoms, proteinuria, plasma and urinary ↑GL3.		(ter). ERT should be started early, in both boys and girls, before irreversible tissue damage.
**2008**	Parini R et al.[60]	Enzyme replacement therapy with agalsidase alfa in a cohort of Italian patients with Anderson-Fabry disease: testing the effects with the Mainz Severity Score Index.	Observational multicentric	3	ALPHA	30 patients with FD, but only 5 boys between 4 and 18 years old.	They evaluated the MSSI index in patients treated with agalsidase alpha.	General and kidney ALPHA ↓MSSI, and prevents CV involvement, justifying its use in the pediatric population. MSSI correlates with age.
**2009**	Phadke SR et al.[61]	Fabry disease: a treatable lysosomal storage disorder.	Case report	5	BETA	A 13-year-old boy with acroparesthesia.	Patient used beta; assessment of neuropathic pain parameters.	ERT ↓neuropathic pain in 4 to 6 months and prevented complications in major organs. 2 symptomatic family members identified by family screening.
**2010**	Zarate YA et al.[62]	A case of minimal change disease in a Fabry patient.	Case report	5	BETA	Boy, 10 years old: neuropathic pain and family history. Evolved with SAH and moderate aortic insufficiency, abdominal pain, worsening kidney function, proteinuria, hypoalbuminemia, and hematic, hyaline casts.	Prominent podocytes and GL3 inclusions with focal tubular atrophy. Cricoid and ERT were started. He recovered kidney function, normalized albuminemia and proteinuria.	BETA + corticosteroid by MHC. Authors speculate: the inflammatory process by FD contributed to MHC. ↓ rapid pain relief and reversal of kidney changes.
**2010**	Schiffmann R et al.[63]	Four-year prospective clinical trial of agalsidase alfa in children with Fabry disease.	Prospective open, extension phase III	1c	ALPHA	17 patients (16 boys), between 7.3 and 18.4 years; 6 months of ALPHA. 10 cases completed the 4-year extension phase.	Baseline and every 6 month-assessment for up to 4 years: plasma and urinary GL3, cardiac changes, GFR, albuminuria, pain, presence of IgG and IgE Ab.	Significant improvement in pain, heart rate, and ↓ plasma and urinaryGL3 with 6 to 12 months of ALPHA, sustained up to 4 years. GFR normalization of hyperfiltrators and in the case with CKD 2 at baseline. The Ab IgG rate was low and no IgE Ab.
**2010**	Park KB et al.[64]	Early diagnosis of Fabry disease in a patient with toe tip pain.	Case report	5	BETA	A 13-year-old boy with intractable neuropathic pain and anhidrosis.	Observe clinical improvement of pain, including ↓ of medications.	Consistent pain improvement with BETA, with no adverse effects up to 4 hours after infusion.

Year	Author (s)	Title	Study type	* Level of evidence	ERT	Patients	Methods	Conclusions
**2010**	Martin-Suárez I et al.[65]	Beneficial effects of long-term enzyme replacement therapy in a child with Fabry disease.	Case report	5	ALPHA	Boy, 15 years old, GI symptoms since 3 years old; anhidrosis, heat intolerance, angiokeratomas and cornea verticilatta since 8 years. Diagnosed at age 9 and ALPHA at age 10.	To evaluate the combined effects of symptomatic treatment and ALFA for 5 years. Mother and paternal grandfather diagnosed by screening.	Early treatment onset ↓ GI symptoms. After 5 years: normal kidney and cardiac function and only angiokeratoma and cornea verticilatta remained. No adverse events reported.
**2011**	Kanai T et al.[66]	Foot process effacement with normal urinalysis in classic Fabry disease.	Case report	5	BETA	Boy, 13 years old, acroparesthesia. BIOPSY: podocyte depletion, fusion of the foot processes and mild glomerular hyperfiltration.	BETA for 14 months. Assessment: urinary beta 2 microglobulin and GFR.	Presymptomatic BETA had a good prognosis; after 14 months: normal plasma GL3, normal kidney function, normal urinalysis, and no other manifestations of the disease. No adverse events. was not rebiopsied.
**2011**	Ramaswami U et al.[67]	Safety of agalsidase alfa in patients with Fabry disease under 7 years.	Retrospective register series	3	ALPHA	8 children < 7 years (5.0±1.6) with neuropathic and/or abdominal pain when starting ALPHA without premedication. Early familial nephropathy was decisive for initiating ALPHA.	Assessment: Estimated GFR, echocardiogram, infusion reactions and presence of anti-ALFA IgG Ab. Treatment time: 1.2 to 6.7 years (mean = 4.2).	1st report of ERT in < 7 years. Normal GFR at baseline and maintained. LVMi > 75th percentile in 5/6 children at baseline. At the end of the study, only 2 with LVMi > normal limit. Anti-alpha IgG antibody in 1 case that had 2 mild and 1 moderate reactions.
**2012**	Ramaswami U et al.[68]	Fabry disease in children and response to enzyme replacement therapy: results from the Fabry Outcome Survey.	Register data	3	ALPHA	FOS patients, 64 boys and 34 girls, who started ALFA below 18 years and treated for 6 ± 3 months.	Assessment: GFR, general symptoms, GI symptoms, pain, proteinuria.	1 to 2 years of ALPHA: significant ↓ pain, GI symptoms and weight and height Z score; Stable eGFR, progressive ↓ number of cases with microalbuminuria.
**2013**	Tøndel C et al.[69]	Agalsidase benefits renal histology in young patients with Fabry disease.	Prospective, comparing ALPHA and BETA	1a	ALPHA and BETA	12 patients (8 to 18 years old). 5 years of ALPHA or BETA, with dose and ERT change. Glomerular hyperfiltration zero cases, 6 with microalbuminuria, 1 with proteinuria and ↑ plasma and urinary GL3 in 7.	6 cases with microalbuminuria, 1 with proteinuria and ↑ plasma and urinary GL3 in 7 cases. Renal biopsies at baseline and after 5 years of ERT.	Normal GFR, ↓plasma and urinary GL3 in all cases; in 5/11 of the cases microalbuminuria disappeared, only 1 ↑ proteinuria (glomerular and tubular proteinuria). ↓ GL3 in mesangial and endothelial cells in all, and it disappeared in tubular epithelial cells in 2 cases, ↓ DS3 in all but 1 case with refractory bradycardia (2 doses of alpha and then beta). In Pediatricians, it is

Year	Author (s)	Title	Study type	* Level of evidence	ERT	Patients	Methods	Conclusions
								difficult to conclude that one drug is better than the other. The biggest difference is perhaps the age of onset.
2013	Lynch M et al.[70]	Fabry's disease in a female, still an under-recognized disease.	Case report	5	Unspecified ERT	Wheelchair girl, 9 years old, acroparesthesias. angiokeratoma, cornea verticilatta and GL3 on endothelial cells.	Unspecified ERT	significant pain ↓and improvement in QoL. She did not repeat the renal biopsy.
2013	Havranek S et al.[71]	Early cardiac changes in children with Anderson Fabry disease.	Observational Retrospective	3	ALPHA	22 patients (11 boys), median age = 9.8 years (2.5 to 16). 7/22 (32%) patients (5 boys) received ALPH from 2005 to 2011.	Assessment: EKG, echocardiogram. 5 boys received ALPHA, 1 case (11 years old) with T wave inversion and his brother (6 years old) with LVH and T wave alteration.	Early and subclinical cardiac changes. EKG changes in children may go unnoticed. Patients on ERT can develop LVH. Heart disease may be less responsive to ERT depletion of deposits. Emphasizes the importance of early ERT.
2013	Borgwardt L et al.[72]	Fabry disease in children: agalsidase-beta enzyme replacement therapy.	National Retrospective Cohort	3	BETA	10 children (9-16 years), 6 boys and 4 girls.	8-year monitoring. Assessment: neuropathic pain, GI symptoms, GFR (estimated and measured), kidney ultrasound, brain MRI, audiometry, albuminuria and proteinuria, EKG, echocardiogram, plasma and urinary GL3, and sweat test.	↓Plasma GL3. 1 boy normalized albumin/creatinine. ↓ acroparesthesias, abdominal pain, ↑energy and physical activity. The authors conclude that, although not all changes revert with BETA, such as cardiac changes, which are even the most difficult to reverse, significant improvement already indicates early treatment. In case of persistent infusion reaction with BETA despite premedication, switch to ALFA.
2013	Furujo M et al.[73]	Enzyme replacement therapy in two Japanese siblings with Fabry disease, and its effectiveness on angiokeratoma and neuropathic pain.	Case report	5	ALPHA	Sibling 1: 13 years old, angiokeratomas, pain, symptoms of dysautonomia, ↓ height and weight, ↑ plasma and urinary GL3. Sibling 2: 11 years old, detected by family screening, hypohidrosis, cornea	Follow-up for 5 years evaluating the effectiveness of ERT with ALPHA.	↓ pain, angiokeratoma, glomerular hyperfiltration, plasma and urinary GL3. No new manifestations in 5 years. No infusion reaction and no IgG Ab against alpha.

Year	Author (s)	Title	Study type	* Level of evidence	ERT	Patients	Methods	Conclusions
						verticilatta, plasma and ↑urinary GL3.		
**2013**	Manwaring V et al.[74]	The identification of new biomarkers for identifying and monitoring kidney disease and their translation into a rapid mass spectrometry-based test: evidence of Presymptomatic kidney disease in pediatric Fabry and type-I diabetic patients.	Prospective cohort	2	Unspecified ERT	10 boys, between 6 and 16 years (mean = 12.5), ↑ urinary GL3. No changes in GFR and proteinuria.	Pre and 12-month urinary proteomics and GL3 study with ERT. Typical responders (n=2) ↓ urinary GL3 and atypical responders: ↑ GL3 (n=2).	↓ GL3: 2x ↑ in GM2 ganglioside (GM2AP) pre and ↓after 1 year of ERT. GM2AP →potential marker. GM2AP collaborates in lipid transport and binds to platelet activating factor (PAF), which is associated with myocardial infarction, stroke.
**2013**	Rombach SM et al.[75]	Long term enzyme replacement therapy for Fabry disease: effectiveness on kidney, heart and brain.	Series of cases included in a Cohort study	4	ALPHA & BETA	2 boys and 4 girls (15.9 to 17.7 years), median follow-up = 4.7 years, acroparesthesia, microalbuminuria, white matter lesion (WML).	Assessment: LV mass, WML, GFR (MDRD or Schwartz), every 3 or 6 months; microalbuminuria and proteinuria.	In the group of adolescents, ERT promoted stabilization of eGFR, with glomerular hyperfiltration normalization; the cardiac part remained stable; 1 case of new WML and 1 patient with WML at baseline had asymptomatic lacunar infarction. The longer the treatment, the fewer complications occur.
**2014**	Nishida M et al.[76]	A case of Fabry nephropathy with histological features of oligonephropathy.	Case report	5	Unspecified ERT	Boy, 13 years old, 27 weeks premature, carrier of late-onset mutation with early worsening of renal function.	↑ serum creatinine, cystatin and serum uric acid, mild proteinuria. No heart changes or other symptoms/signs.	Conditions such as prematurity and low birth weight can lead to oligonephropathy and catalyze the onset of Fabry nephropathy. Early treatment of SCD stabilized renal function and ↓ proteinuria. SCD mother with ↓AGAL-A activity and ↑lyso-GL3.
**2014**	Schiffmann R et al.[77]	Agalsidase alfa in pediatric patients with Fabry disease: a 6.5-year open-label follow-up study.	Outreach study (6.5 years) multicentric open	1c	ALPHA	17 patients (10 boys and 1 girl from phase 1, baseline, age 8.6–17.3 years).	Phase 2: after 4 years of phase 1 and the average time evaluated = 6.5 ± 0.6 years. Assess: safety, cardiac parameters, eGFR, proteinuria, plasma GL3 and pain.	ALPHA well tolerated, mild or moderate adverse events, which ↓ with time of treatment. ↓Ac antialpha and did not influence ERT response. The eGFR was normal, proteinuria was stable and < 0.2 in all patients. 1 patient had anti-alpha neutralizing

Year	Author (s)	Title	Study type	* Level of evidence	ERT	Patients	Methods	Conclusions
								Abs and was the only one with slightly increased urinary GL3 at the end compared to baseline. Agalsidase alfa may be a useful therapeutic option started early and maintained for a long time in these patients.
**2014**	Lin HY et al.[78]	Clinical observations on enzyme replacement therapy in patients with Fabry disease and the switch from agalsidase beta to agalsidase alpha.	Report of 1 adolescent included in a case series	5	ALPHA & BETA	One 14-year-old with fever and chills.	Observation of FD markers during ERT started at age 14.	Safe ERT, good when early. This patient had hyperfiltration. ↓ LMVi. No difference between alpha and beta after switching from beta to alpha.
**2014**	Iemolo F et al.[79]	De novo mutation in a male patient with Fabry disease: a case report.	Case report	5	Unspecified ERT	Girl, 11 years old, with acroparesthesias, GI symptoms, heat intolerance and proteinuria. Father, 44 years old, with severe FD involvement, undergoing kidney transplantation.	Assessment: clinical after initiation of UNSPECIFIED ERT.	Father only improved from acroparesthesias and angiokeratomas. The daughter improved in all her symptoms. This report emphasizes the importance of early having and family screening.
**2014**	Anderson LJ, et al.[80]	Long-term effectiveness of enzyme replacement therapy in Fabry disease: results from the NCS-LSD cohort study.	Cohort	3	ALPHA & BETA	22 cases, 11 boys. Earlier diagnosis in boys. Median 1st infusion = 9.7 years (8.5 to 11.3). At recruitment: 3 boys with beta and 1 with alpha; 2 girls with alpha and 1 with beta. Normal eGFR, less in 1 boy (15 years) with ↓↓GFR.	Assessment: cognitive aspects, social adaptation, QoL, LV mass index, proteinuria, eGFR, pain, hearing and transient ischemic attacks. ALFA and BETA analyzed together.	There was no change in any of the parameters. However, the children were oligo or asymptomatic.
**2015**	Bugescu N et al.[81]	The neurocognitive impact of Fabry disease on pediatric patients.	Cohort with and without ERT	1b	BETA	24 patients (10 boys and 14 girls; 6 to 18 years), 7 (35% had BETA ERT at baseline and 13 (65%) had no ERT.	Assessment: family history, demographics, and neurocognitive function. Rating scale: 1 (never), 2 (sometimes) and 3 (often).	Criteria under ERT had more ↑ cognitive function scores and ↓ symptoms of inattention, memory and executive function problems. No significant difference in hyperactivity/impulsivity. This study warns about the importance of early ERT.
**2015**	Tøndel C et al.[82]	Foot process effacement is an early marker of	Case series	3	ALPHA	6 boys and 2 girls, median age = 12 years (4-16) with	GFR (Iohexol and/or eGFR Plasma Clearance) AND	Study showed ↓ pain, ↓ GL3 deposits, clearance in other cells and

Year	Author (s)	Title	Study type	* Level of evidence	ERT	Patients	Methods	Conclusions
		nephropathy in young classic Fabry patients without albuminuria.				normal neuropathic pain, albuminuria and eGFR. 3 re-biopsies after 3 years (1 girl aged 12 years at baseline) and after 5 years (2 boys, 12 and 16 years at baseline).	Albuminuria. pre-ERT: all with podocyte deposit of GL3 and other cells.	↑podocyte fusion in 3 children. Suggests that renal biopsy is essential for staging renal involvement, including re-biopsy.
**2016**	Kim JH et al.[83]	Long-term enzyme replacement therapy for Fabry disease: efficacy and unmet needs in cardiac and renal outcomes.	Series with 19 patients (4 children)	4	BETA	19 patients, 4 children (3 family screening) males with neuropathic pain, normal kidney function, zero proteinuria. 1 boy with Wolff-Parkinson-White syndrome. Normal dose of beta and ↓ for 23 months.	Evaluate the effects of BETA for at least 5 years. Rebiopsy in 3:1 global glomerular sclerosis. Monitoring, every 3 to 6 months: clinical parameters, serum creatinine, eGFR (MDRD/Schwartz), 24-hour proteinuria, plasma and urinary GL3.	Normal kidney function and no proteinuria were maintained; 1 patient developed bradycardia. No cerebrovascular events. Histological kidney change happened before functional changes. They emphasize the importance of kidney histology.
**2016**	Ito S et al.[84]	Significant improvement in Fabry disease podocytopathy after 3 years of treatment with agalsidase beta.	Previously published case report with longer follow-up time	5	BETA	Clinical course and kidney histology of a 10-year-old boy before and after 3 years of BETA.	Vacuolization, GL3 inclusions in podocytes and their fusion. Normal dose BETA with no change, but with low dose had proteinuria.	Rebiopsy after 3 years of BETA: significantly ↓vacuolization and GL3 inclusions. There was no podocyte fusion. It shows the importance of histology in the follow-up of Fabry nephropathy.
**2016**	Politei J et al.[85]	Gastrointestinal involvement in Fabry disease. So important, yet often neglected.	Report of a pediatric case included in a series of 4 cases	4	BETA	Boy, 17 years old: neuropathic pain, hypohidrosis, heat intolerance, angiokeratomas and cornea verticilatta since age 12 and abdominal pain and diarrhea since age 13. Detected by family screening.	BETA started at age 17. Assessment of pain parameters and GI symptoms.	After 1 year of BETA significantly↓ GI symptoms and neuropathic pain.
**2016**	Kanai T et al.[86]	Surges in proteinuria are associated with plasma GL3 elevations in a young patient with classic Fabry disease.	Case report	5	BETA	A 13-year-old boy with acroparesthesias, ↑ plasma GL3, normal renal function, no proteinuria, diffuse GL3 deposits in podocytes and segmental podocyte fusion.	Follow-up during ↓ BETA stock, received ↓ dose. Evaluation of plasma GL3, proteinuria and renal histology.	With ↓ BETA dose: ↑ proteinuria and ↑ GL3, reversed after dose normalization. Final kidney histology: podocytes practically free of deposits. Recommended BETA dose promotes proteinuria improvement and normal plasma GL3. Transient IgG antibodies against BETA at treatment onset.

Year	Author (s)	Title	Study type	* Level of evidence	ERT	Patients	Methods	Conclusions
**2016**	Goker-Alpan O et al.[87]	An open-label clinical trial of agalsidase alfa enzyme replacement therapy in children with Fabry disease who are naïve to enzyme replacement therapy.	Phase II open multicenter	1c	ALPHA	14 children (7 to 18 years) received ALPHA for 54.5 weeks (54 - 59). 5 boys aged between 6.7 and 14.4 years and 9 girls aged between 10.1 and 15.9 years.	Pain assessment (BPI), cardiac involvement, plasma and urinary GL3, GFR, proteinuria, microalbuminuria and kidney histology.	ALPHA ↓ pain, normal heart rhythm, ↓ tissue deposits of GL3, maintaining normal eGFR and without proteinuria with deposits clearance in kidney tissue. A boy with neutralizing Ab and maintained efficacy parameters.
**2017**	Skrunes R et al.[43]	Long-Term dose-dependent Agalsidase effects on kidney histology in Fabry disease.	Observational Cohort	3	Unspecified ERT	20 patients, 10 of which started ERT with < 18 years, 6 received a higher dose. It does not specify the enzyme used per case.	Clinical and kidney histology follow-up for 14 years.	Dose-dependent GL3 clearance in podocytes. Suggests that the dose should be individualized.
**2017**	Skrunes R et al.[88]	Reaccumulation of globotriaosylceramide in podocytes after agalsidase dose reduction in young Fabry patients.	Case series	4	ALPHA & BETA	3 patients started BETA, median = 11 years (7 to 18 years); after 5 years, switch to ALPHA and reassessment after 3 years.	Kidney biopsy after 5 years of BETA and after 3 years of ALPHA. Assessment: GL3 deposits. 1 patient rebiopsied after 2 years of return from ALPHA to BETA.	↑ GL3 podocyte deposits after switch from BETA to ALPHA. Can rebiopsy be useful for treatment choice? At month 2:1 patient developed IgG anti beta antibodies which disappeared at month 25.
**2019**	Ramaswami U et al.[89]	Low-dose agalsidase beta treatment in male pediatric patients with Fabry disease: A 5-year randomized controlled trial.	Phase III multicenter randomized open-label, 2 groups receiving BETA: low dose vs normal dose.	1a	BETA	31 boys, mean age = 11.6 years (ranging from 5 to 18), with no clinical evidence of cardiac, kidney or cerebral involvement.	Low dose every 2 weeks and normal dose every 4 weeks, 27 cases completed the study. Sequential skin biopsies: GL3 in superficial endothelial capillaries.	Deposit clearance was lower in all cases with lower dose. Low-dose regimens do not provide the benefit in reducing deposits like the recommended regimen.
**2019**	Sasa H et al.[90]	Safety and effectiveness of enzyme replacement therapy with agalsidase alfa in patients with Fabry disease: Post-marketing surveillance in Japan.	Post-marketing surveillance. Series of 4 cases < 10 years inserted in Cohort.	3	ALPHA	Long-term follow-up of patients receiving alpha, 4 patients < 10 years.	Clinical, biochemical and kidney histology follow-up.	↑ plasma GL3 after 1 year of ALPHA, but it decreased after 3 years. GFR loss was smaller the higher it was at baseline. This study shows the safety and efficacy of early ALPHA.
**2019**	Madsen CV et al.[91]	Age-related renal function decline in Fabry disease patients on enzyme replacement therapy: a longitudinal cohort study.	Cohort	3	ALPHA & BETA	52 cases; 62% women. Age between 9 and 67 years, median = 33.	Patients < 18 years: measured GFR. ERT alpha and beta analyzed together.	Urinary protein/creatinine > 300 mg/g at baseline was critical for faster ↓ GFR. Plasma GL3 normal, but lyso-GL3 ↑ in 34% of cases. Better kidney function is associated with earlier onset of ALPHA and ↓

Year	Author (s)	Title	Study type	* Level of evidence	ERT	Patients	Methods	Conclusions
								baseline proteinuria. Lyso-GL3 may remain ↑ in a % of cases.
**2020**	Parini R et al.[92]	Analysis of renal and cardiac outcomes in male participants in the Fabry Outcome Survey starting Agalsidase Alfa enzyme replacement therapy before and after 18 years of age.	Retrospective with register data	3	ALPHA	ALPHA age of onset: - cohort 1 (children): ≤18 years old (n=151) - cohort 2 (young adults): >18 and ≤30 years old (n=155) and cohort 3 (adults): >30 years (n=254). long follow-up time.	Assessment: MSSI-FOS index, GFR (Counahan-Barrat and CKD-EPI), proteinuria, LV mass index (>50 g/m2SC → LVH).	Early ALPH promotes mitigation of kidney and cardiac manifestations, and the earlier the result was more expressive.
